# Rapid elevation of sodium transport through insulin is mediated by AKT in alveolar cells

**DOI:** 10.1002/phy2.269

**Published:** 2014-03-20

**Authors:** Charlott Mattes, Mandy Laube, Ulrich H. Thome

**Affiliations:** ^1^ Division of Neonatology Center for Pediatric Research Leipzig Hospital for Children & Adolescents University of Leipzig Leipzig 04103 Germany

**Keywords:** AKT, alveolar cells, epithelial Na^+^ channel (ENaC), insulin, SGK1

## Abstract

Alveolar fluid clearance is driven by vectorial Na^+^ transport and promotes postnatal lung adaptation. The effect of insulin on alveolar epithelial Na^+^ transport was studied in isolated alveolar cells from 18–19‐day gestational age rat fetuses. Equivalent short‐circuit currents (*I*_SC_) were measured in Ussing chambers and different kinase inhibitors were used to determine the pathway of insulin stimulation. In Western Blot measurements the activation of mediators stimulated by insulin was analyzed. The *I*_SC_ showed a fast dose‐dependent increase by insulin, which could be attributed to an increased ENaC (epithelial Na^+^ channel) activity in experiments with permeabilized apical or basolateral membrane. 5‐(N‐Ethyl‐N‐isopropyl)amiloride inhibition of *I*_SC_ was not affected, however, benzamil‐sensitive *I*_SC_ was increased in insulin‐stimulated monolayers. The application of LY‐294002 and Akti1/2 both completely blocked the stimulating effect of insulin on *I*_SC_. PP242 partly blocked the effect of insulin, whereas Rapamycin evoked no inhibition. Western Blot measurements revealed an increased phosphorylation of AKT after insulin stimulation. SGK1 activity was also increased by insulin as shown by Western Blot of pNDRG1. However, in Ussing chamber measurements, GSK650394, an inhibitor of SGK1 did not prevent the increase in *I*_SC_ induced by insulin. The application of IGF‐1 mimicked the effect of insulin and increased the ENaC activity. In addition, an increased autophosphorylation of the IGF‐1R/IR was observed after insulin stimulation. We conclude that insulin rapidly increases epithelial Na^+^ transport by enhancing the activity of endogenous ENaC through activation of PI3K/AKT in alveolar cells.

## Introduction

During postnatal lung adaptation alveolar fluid has to be removed to promote air breathing. Alveolar fluid clearance (AFC) is driven by unidirectional Na^+^ transport through pneumocytes. Na^+^ enters the cells through epithelial sodium channels (ENaC) in the apical plasma membrane and is actively extruded through the basolateral membrane by Na,K‐ATPases. Both transporters are essential for vectorial Na^+^ transport, although the uptake of Na^+^ by ENaC is usually rate‐limiting (O'Brodovich et al. 1990; Hummler et al. 1996). Impaired AFC leads to wet lung syndrome and respiratory distress syndrome (RDS; Helve et al. 2004). In preterm infants, these morbidities occur more frequently and studies showed that smaller amounts of ENaC are expressed, suggesting a dependency of AFC on ENaC function (Helve et al. 2004; Janer et al. 2010). Knockout of *α*‐ENaC (Hummler et al. 1996) or inhibition of ENaC‐mediated Na^+^ currents (O'Brodovich et al. 1990) leads to RDS and lung failure. Therefore, lung edema in at‐risk subjects may be prevented by increasing the function of ENaC or Na,K‐ATPases, which could be particularly beneficial for preterm infants.

Since the function of ENaC is crucial for water and Na^+^ homeostasis, it is distinctively regulated at different cellular levels. Hormones like female sex steroids (Laube et al. 2011), aldosterone (Lee et al. 2008), glucocorticoids (Lazrak et al. 2000; Inglis et al. 2009) or insulin (Hagiwara et al. 1992; Lee et al. 2008; Deng et al. 2012) are known to increase ENaC function. In renal cells, a stimulating effect of insulin on Na^+^ transport was demonstrated through phosphorylation of ubiquitin ligase Nedd4‐2 (neural precursor cell expressed, developmentally downregulated protein 4‐2; Lee et al. 2008), enhanced ENaC trafficking (Tiwari et al. 2007) or direct modification of ENaC molecules (Shimkets et al. 1998; Zhang et al. 2005; Diakov et al. 2010). The serum‐ and glucocorticoid‐regulated kinase 1 (SGK1; Murray et al. 2005; Shimkets et al. 1998), AKT (protein kinase B; Diakov et al. 2010; Lee et al. 2007), mammalian target of rapamycin complex 1 (mTORC1; Proud 2007) and mTORC2 (Mansley and Wilson 2010a) were identified as possible mediators of ENaC stimulation. Nevertheless, knowledge about the effects of insulin on Na^+^ transport in respiratory epithelia is limited. Some clinical studies suggest that insulin stimulates AFC and improves lung function in adults (Guazzi et al. 2002a,b) and insulin was found to improve AFC and outcome in a model of ALI in mice (Deng et al. 2012). Therefore, the goal of this study was to determine the onset of insulin stimulation on Na^+^ transport and to identify the mediators of this effect on endogenous ENaC in alveolar cells of fetal origin, because these cells presumably serve as a better model than adult alveolar cells for processes during fetal/neonatal transition. Thereby, we aimed to identify possible targets to pharmacologically stimulate Na^+^ transport, which may be very beneficial in patients at birth with severe acute lung disease.

## Methods

### Cell isolation and cell culture

All animal care and experimental procedures were approved by the responsible authority (Landesdirektion Leipzig). Sprague‐Dawley rats were bred at the Medical Experimental Center (MEZ) of the University of Leipzig. The animals were housed in rooms with a controlled temperature (22°C), humidity (55%), and 12‐h light‐dark cycle. Food and water were freely available. The pregnant rats were euthanized by carbon dioxide inhalation.

Fetal distal lung epithelial (FDLE) cells, a model of preterm respiratory cells, were isolated as described previously (Jassal et al. 1991; Thome et al. 2003). In brief, lungs were minced and digested in a solution with 0.125% trypsin (Life technologies, Darmstadt, Germany) and 0.4 mg/mL DNAse (CellSystems, Troisdorf, Germany) in MEM (Biochrom, Berlin, Germany) for 10 min at 37°C. The digestion was stopped by adding MEM containing 10% FBS (PAA Laboratories, Cölbe, Germany). The cells were centrifuged (440 ***g***) and resuspended in 15 mL MEM containing 0.1% collagenase (CellSystems) and DNAse for further digestion. The solution was incubated for 15 min at 37°C. The collagenase activity was stopped by adding 15 mL MEM containing 10% FBS. Cells were plated twice for 1.5 h to remove contaminating fibroblasts. The supernatant contained epithelial cells with >95% purity (Jassal et al. 1991). For Ussing chamber measurements, cells were seeded on Snapwell permeable supports (Costar^®^ No. 3407, Inc., Corning, NY, 1 cm^2^) at a density of 10^6^ cells per insert. For Western Blot measurements cells were seeded on Transwell permeable supports (Costar^®^ No. 3412, 2.4 cm^2^) at a density of 2 × 10^6^ cells per insert. The culture medium, containing MEM with 10% FBS, 2 mmol/L L‐glutamine (PAA Laboratories), and Antibiotic/Antimycotic (Life technologies, containing penicillin [100 units/mL], streptomycin [100 *μ*g/mL] and amphotericin B [0.25 *μ*g/mL] as antibacterial and antifungal supplement), was changed daily. Cells subjected to different experimental conditions were always age matched, derived from the same litter, treated equally, and recorded simultaneously.

### Electrophysiological measurements

A detailed description of Ussing chamber measurement procedures is reported elsewhere (Thome et al. 2003). Experiments took place on the 4th day of culture and were included in the analysis only when the transepithelial resistance (*R*_te_) exceeded 300 Ω cm² throughout the measurement. The Ussing chambers were filled with a solution containing (in mmol/L): Na^+^ 145, K^+^ 5, Ca^2+^1.2, Mg^2+^1.2, Cl^–^ 125, HCO_3_^–^ 25, H_2_PO_4_^−^ 3.3, HPO_4_^2−^ 0.8 (pH 7.4). The basolateral side contained 10 mmol/L glucose whereas 10 mmol/L mannitol was used in the apical compartment. Equivalent short‐circuit currents (*I*_sc_) were assessed every 20 sec by measuring transepithelial voltage (*V*_te_) and resistance (*R*_te_) using a transepithelial current clamp (Physiologic instruments, San Diego, CA), and calculating the quotient *I*_sc_ = *V*_te_/*R*_te_. Δ*I*_SC_ (in *μ*A/cm^2^) was calculated as the difference between currents measured before and after addition of insulin, other compounds, or the respective solvent (control), representing the induced current change or the normal fluctuations of currents during the recording. Insulin in three different concentrations (20 nmol/L, 200 nmol/L, and 2 *μ*mol/L, I‐6634, Sigma, Germany) or IGF‐1 (200 nmol/L, Biozol, Germany) was added to the basolateral compartment of the Ussing chambers to stimulate monolayers. Amiloride (20 *μ*mol/L, A‐7410, Sigma), an inhibitor of ENaC, was added to the apical compartment to determine the amiloride‐sensitive *I*_SC_ (*I*_amil_). Ouabain (1 mmol/L, O‐3125, Sigma), an inhibitor of Na,K‐ATPases, was added to the basolateral compartment to determine the ouabain‐sensitive *I*_SC_ (*I*_ouab_), accordingly. Amiloride was applied after the insulin‐induced *I*_SC_ reached a stable plateau (15–20 min after addition). To measure the current inhibition by benzamil and 5‐(N‐Ethyl‐N‐isopropyl)amiloride (EIPA), 10 *μ*mol/L benzamil (B‐2417, Sigma), or 100 *μ*mol/L EIPA (A‐3085, Sigma) was added to the apical compartment and the antagonist‐sensitive *I*_SC_ was determined. In experiments using kinase inhibitors, the inhibitors were added to both compartments 30 min prior to addition of insulin. LY‐294002 (50 *μ*mol/L, 1130, TOCRIS bioscience, Bristol, UK) was used as an inhibitor of Phosphatidylinositide 3‐kinases (PI3K), GSK650394 (10 *μ*mol/L, 3572, TOCRIS) used as an inhibitor of SGK1, Akti1/2 kinase inhibitor (50 *μ*mol/L, A‐6730, Sigma) as an inhibitor of AKT, Rapamycin (100 nmol/L, 13346, Cayman Chemical Company, Ann Arbor, MI) used as an inhibitor of mTORC1 and PP242 (1 *μ*mol/L, CD0258, Chemdea, Ridgewood, NJ) as an inhibitor of mTORC1 and mTORC2 (Rapamycin and PP242 kindly provided by A. Garten). The employed concentrations were based on previous studies (Barnett et al. 2005; Inglis et al. 2009; Mansley and Wilson 2010b).

In some experiments, the apical or basolateral membrane was permeabilized by addition of 100 *μ*mol/L amphotericin B (A‐4888, Sigma) to the basolateral compartment or 10 *μ*mol/L to the apical compartment (Kirk and Dawson 1983) and *I*_SC_ measured every 5 sec with a transepithelial voltage clamp. Thereby, Na^+^ transporters on either side of the cell can be measured and analyzed separately from the other. For measurements with permeabilized basolateral membrane, a 145:5 mmol/L apical to basolateral Na^+^ gradient was used across the monolayer by replacing 140 mmol/L Na^+^ on the basolateral side with 116 mmol/L *N*‐methyl‐D‐glucamine and 24 mmol/L choline. After permeabilization, Na^+^ flux is passive and solely determined by the permeability of the apical membrane holding ENaC. After the maximal *I*_SC_ was reached by permeabilization of either membrane, amiloride or ouabain was added to determine maximal *I*_amil_ or *I*_ouab_, accordingly.

Differences among groups treated with different substances and controls were evaluated by the unpaired *T*‐test or Mann–Whitney test, depending on data distribution. For the comparison of more than two groups an ANOVA with Dunnett's or Tukey's *post hoc* test was used.

### Western blot measurement

A detailed description can be found elsewhere (Thome et al. 2003). Phosphorylation of the n‐myc downregulated gene 1 (NDRG1) was used as an assay of SGK1 enzyme activity (Murray et al. 2005; Inglis et al. 2009; Wilson et al. 2010) and detected with phospho‐NDRG1 antibody (5482, Cell Signaling Technology, Inc., Danvers, MA) when phosphorylated at Thr346 and NDRG1 antibody (9408, Cell Signaling Technology, Inc.). Phosphorylation of AKT was analyzed using antibodies against phospho‐AKT at Thr308 (4056, Cell Signaling Technology, Inc.), and AKT (9272, Cell Signaling Technology, Inc., both kindly provided by J. Klammt) to analyze the activation of the PI3K/AKT pathway. Thr308 is a phosphorylation site of phosphoinositide‐dependent kinase‐1 (PDK1). Finally, phosphorylation of IGF‐1 receptor/insulin receptor (IGF‐1/IR) was detected using antibodies against phospho‐IGF‐1R*β* (Tyr1135/1136)/IR‐*β* (Tyr1150/1151; 3024, Cell Signaling Technology, Inc.) and IGF‐1R*β* (3027, Cell Signaling Technology, Inc., both kindly provided by J. Klammt). Adjacent lung fibroblasts obtained during cell isolation were used as control cell line in AKT and IGF‐1R/IR Western Blot measurements. The fibroblasts were also seeded on Transwell supports and treated equally. For all Western Blots, FDLE cells were incubated with 200 nmol/L insulin dissolved in serum‐free media (Cellgro, Mediatech, Herndon, VA) for 20 min and compared to control monolayers incubated in serum‐free media without supplements. The SGK1 inhibitor GSK650384 was added 30 min prior to insulin, to mimic the Ussing chamber experimental time course. Suitable secondary antibodies conjugated to horseradish peroxidase (HRP) were used to detect primary antibodies. HRP activity was analyzed by enhanced chemiluminescence (ECL, Amersham, Piscataway, NJ) on X‐ray film and band intensity was measured by densitometry using Image‐J (NIH).

Amiloride, Ouabain, and IGF‐1 were dissolved in water; all other drugs were prepared in DMSO (kinase inhibitors) or 10 mmol/L HCl (insulin) diluted 1:1000 in electrophysiological solution during measurements. In Ussing chamber and Western Blot experiments, the control monolayers were treated with the same concentration of the respective solvent to exclude solvent influences on the evoked responses.

## Results

### Effect of insulin on vectorial Na^+^ transport

**Figure 1:**
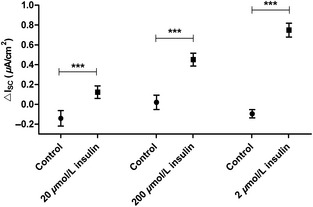
Insulin increases short‐circuit current (*I*_SC_) in a dose‐dependent manner. 20 nmol/L insulin (*n *=* *36 and 42, ***P <* *0.001), 200 nmol/L insulin (*n *=* *32 and 41, ***P <* *0.001) and 2 *μ*mol/L insulin (*n* = 25 and 42, ***P <* *0.001). Mean ± SEM.

**Figure 2:**
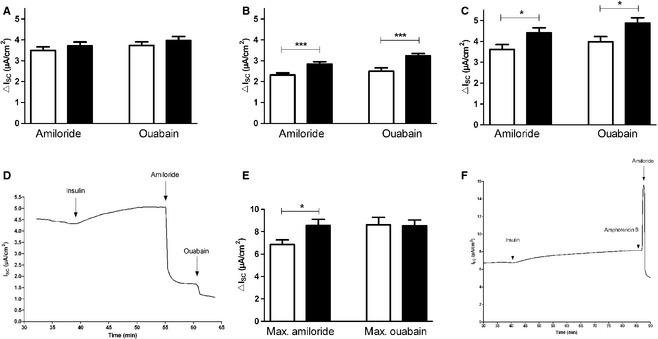
Insulin increases *I*_amil_ and *I*_ouab._ Amiloride (20 *μ*mol/L) and ouabain (1 mmol/L) were added to Ussing chambers after addition of insulin. (A) 20 nmol/L insulin (*n *=* *36 and 42). (B) 200 nmol/L (*n *=* *32 and 41, ***P <* *0.001) and (C) 2 *μ*mol/L insulin (*n *=* *25 and 42, *P <* *0.05). Mean + SEM. (D) Typical tracing of Ussing chamber measurement. After the basal current reached a plateau, insulin (2 *μ*mol/L) was applied. Amiloride (20 *μ*mol/L) was added apically after the insulin response remained constant. Ouabain (1 mmol/L) was applied afterward to the basolateral compartment. (E) Effect of insulin on maximal *I*_amil_ and maximal *I*_ouab_ compared to controls. Amphotericin B was used to permeabilize either the basolateral membrane (100 *μ*mol/L) or the apical membrane (10 *μ*mol/L). The maximal *I*_amil_ represents the current reduction caused by amiloride (20 *μ*mol/L) after permeabilization (*n *=* *16 and 30, *P <* *0.05) whereas the maximal *I*_ouab_ represents the current reduction caused by ouabain (1 mmol/L) after permeabilization (*n *=* *11 and 19). Mean + SEM. (F) Tracing of Ussing chamber measurement. After the basal current reached a plateau, insulin (200 nmol/L) was applied. Amphotericin B (100 *μ*mol/L) was added basolaterally to permeabilize the basolateral membrane and amiloride (20 *μ*mol/L) was applied apically after maximal current increase. 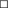, control; 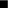, insulin.

**Figure 3:**
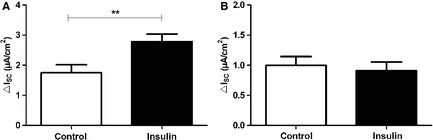
Insulin enhances benzamil‐sensitive *I*_SC__._ Effects of insulin (200 nmol/L) on benzamil‐ and EIPA‐sensitive *I*_SC_ of FDLE cell monolayers. (A) 10 *μ*mol/L benzamil (*n *=* *24 and 25, **P <* *0.01) and (B) 100 *μ*mol/L EIPA (*n *=* *23 and 25). Mean + SEM. 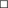, control; 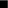, insulin.

All monolayers used in the electrophysiological studies were obtained from 27 different cell isolations. Of 681 monolayers, 670 had an *R*_te_ > 300 Ω cm² and were included in the analysis, their mean *R*_te_ was 1201 ± 414 Ω cm² (Mean ± SD). Insulin significantly increased *I*_SC_ within a few minutes after addition in a concentration‐dependent manner (Fig. 1, *P* < 0.001 by Mann–Whitney test). The electrophysiological measurements showed that insulin raised the basal current by 10–15%. Focusing on the rapid effects of insulin, amiloride was applied after the insulin‐induced *I*_SC_ reached a plateau within 15–20 min. Therefore, possible effects of insulin occurring thereafter were not addressed. *I*_amil_ and *I*_ouab_ were significantly higher in monolayers pretreated with 200 nmol/L insulin (*I*_amil_ and *I*_ouab_, *P *<* *0.001 by *T*‐test, Fig. 2B) and 2 *μ*mol/L insulin (*I*_amil_ and *I*_ouab_, *P *<* *0.05 by *T*‐test, Fig. 2C) compared to controls, whereas only a trend toward an increased *I*_amil_ and *I*_ouab_ was observed after addition of 20 nmol/L insulin (Fig. 2A). A current tracing demonstrating the *I*_SC_ increase induced by insulin and the decrease induced by amiloride and ouabain is shown in Fig. 2D. A robust effect of insulin was seen for 200 nmol/L and 2 *μ*mol/L and, therefore, we chose to use the lower insulin concentration of 200 nmol/L for further analysis. Monolayers stimulated with insulin before permeabilization of the basolateral membrane showed an increased maximal *I*_amil_ (*P *<* *0.05 by *T*‐test, Fig. 2E), representing an increased maximal ENaC activity. Figure 2F demonstrates a current tracing obtained with permeabilized basolateral membrane. After permeabilization of the apical plasma membrane maximal *I*_ouab_ was measured (Fig. 2E), which did not differ from control monolayers. In summary, the experiments showed that insulin rapidly increases *I*_SC_ in FDLE monolayers. These changes were accompanied by elevated *I*_amil_ and *I*_ouab_. In permeabilized monolayers, current increases induced by insulin were only seen with permeabilized basolateral membrane, indicating that insulin increased the permeability of the apical membrane only. To determine how different amiloride‐sensitive Na^+^ channels participate in the insulin‐induced current increase, blocking experiments were carried out using the antagonists EIPA and benzamil. EIPA is known to block nonselective Na^+^ channels whereas benzamil inhibits highly selective Na^+^ channels. The results showed no difference of EIPA‐sensitive *I*_SC_ between insulin‐stimulated and control monolayers (Fig. 3B). However, the benzamil‐sensitive *I*_SC_ was significantly increased in insulin‐stimulated monolayers (*P *<* *0.01 by *T*‐test, Fig. 3A).

### Intracellular mediators in the insulin pathway

**Figure 4:**
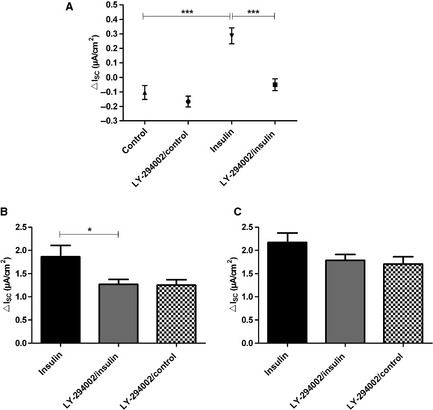
Inhibition of PI3K suppresses the effect of insulin on *I*_SC_. LY‐294002 (50 *μ*mol/L) was added 30 min prior to addition of 200 nmol/L insulin to both compartments (*n *=* *17). For comparison 200 nmol/L insulin without LY‐294002 (*n *=* *12) and unstimulated control monolayers with LY‐294002 (*n *=* *8) were used as control. (A) insulin‐induced Δ*I*_SC_. Mean ± SEM, ***P <* *0.001. (B) *I*_amil_, *P <* *0.05. (C) *I*_ouab_. Mean + SEM. 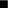, insulin; 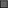, insulin + LY‐294002; 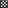, control + LY‐294002.

Inhibition of PI3K by incubation with LY‐294002 (50 *μ*mol/L) for 30 min prior to the addition of 200 nmol/L insulin prevented the stimulatory effect of insulin on *I*_SC_. This is shown by the significantly reduced Δ*I*_SC_ of LY‐294002‐/insulin‐treated monolayers compared to monolayers stimulated with insulin without the inhibitor (*P *<* *0.001 by Tukey's *post hoc* test; Fig. 4A). Monolayers treated with LY‐294002 alone, as additional control, did not differ from LY‐294002‐/insulin‐treated monolayers. The analysis of *I*_amil_ and *I*_ouab_ showed that currents of LY‐294002/control and LY‐294002‐/insulin‐treated monolayers were almost identical, whereas the monolayers treated with insulin alone had increased currents, as shown before (*P *<* *0.05 for *I*_amil_ by Dunnett's *post hoc* test, Fig. 4B and C). These experiments showed that the activity of the PI3K is necessary for the stimulatory effect of insulin on epithelial Na^+^ transport in alveolar cells.

**Figure 5:**
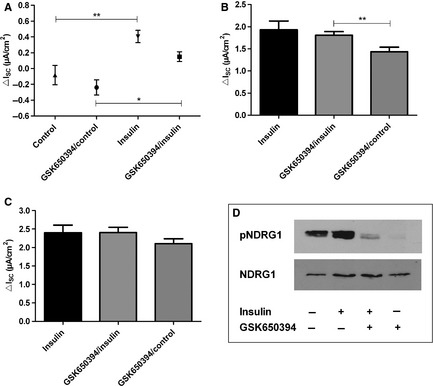
Inhibition of SGK1 did not affect insulin‐induced *I*_SC_ increase. GSK650394 (10 *μ*mol/L) was added 30 min prior to addition of 200 nmol/L insulin to both compartments (*n *=* *13). For comparison 200 nmol/L insulin without GSK650394 (*n *=* *11) and unstimulated control monolayers with GSK650394 (*n *=* *10) were used as control. (A) insulin‐induced Δ*I*_SC_. Mean ± SEM, **P <* *0.01, *P <* *0.05. (B) *I*_amil_, **P <* *0.01. (C) *I*_ouab_. Mean + SEM. (D) Western Blot of pNDRG1 and total NDRG1 in FDLE cells after stimulation with 200 nmol/L insulin compared to unstimulated control and inhibited with GSK650394. 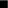, insulin; 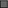, insulin + GSK650394; 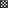, control + GSK650394.

Inhibition of the SGK1 by incubation of monolayers with GSK650394 (10 *μ*mol/L) for 30 min prior to the addition of 200 nmol/L insulin did not prevent the stimulatory effect of insulin on *I*_SC_ (Fig. 5). The GSK650394‐treated monolayers still showed a significant increase in *I*_SC_ after the addition of insulin (*P *<* *0.05 by Tukey's *post hoc* test; Fig. 5A). The *I*_amil_ of GSK650394‐treated FDLE cells was also significantly higher in insulin‐stimulated monolayers (*P *<* *0.01 by Tukey's *post hoc* test; Fig. 5B). The results showed that in the presence of SGK1‐inhibition, insulin was still able to increase Na^+^ transport and thus suggest that SGK1 does not play an important part in rapid insulin stimulation of FDLE cell Na^+^ transport. On the other hand, SGK1 was activated in the cells since the phosphorylation of NDRG1, which is a specific substrate of SGK1, was increased in insulin‐stimulated monolayers compared with controls, as shown by Western Blot (Fig. 5D). Therefore, these results do support an activation of SGK1 by insulin. However, the Western Blot experiments also showed that GSK650394 suppressed the activation of SGK1 by insulin as seen in the blocked phosphorylation of NDRG1 (Fig. 5D). Since the insulin effect in Ussing chamber measurements persisted after application of GSK650394, the activity of SGK1 is not decisively involved in Na^+^ transport regulation of FDLE cells.

**Figure 6:**
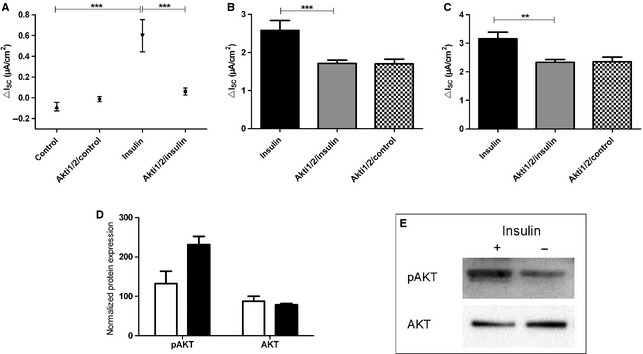
Inhibition of AKT suppresses the effect of insulin on *I*_SC_. Akti1/2 (50 *μ*mol/L) was added 30 min prior to addition of 200 nmol/L insulin to both compartments (*n *=* *17). For comparison 200 nmol/L insulin without Akti1/2 (*n *=* *11) and unstimulated control monolayers with Akti1/2 (*n *=* *12) were used as control. (A) insulin‐induced Δ*I*_SC_. Mean ± SEM, ***P <* *0.001. (B) *I*_amil_, ***P <* *0.001. (C) *I*_ouab_, **P <* *0.01. Mean + SEM. (D) and (E) Western Blot of AKT and phosphorylated AKT in FDLE cells grown in the presence of 200 nmol/L insulin compared to unstimulated controls. (D) Normalized densitometric evaluation of pAKT and AKT. (E) Western Blot of pAKT and total AKT resulted in 60 kDa bands (*n *=* *3). 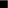, insulin; 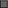, insulin + Akti1/2; 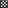, control + Akti1/2; 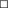, control.

Next, we blocked AKT by adding 50 *μ*mol/L Akti1/2 kinase inhibitor to Ussing chambers 30 min prior to adding 200 nmol/L insulin (Fig. 6). Akti1/2 treatment completely abolished the stimulating effect of insulin on *I*_SC_. This is demonstrated by the significantly lower Δ*I*_SC_ of Akti1/2/insulin‐treated monolayers compared to cells stimulated with insulin alone (*P *<* *0.001 by Tukey's *post hoc* test; Fig. 6A). Furthermore, *I*_amil_ and *I*_ouab_ observed after addition of insulin were also significantly decreased by Akti1/2 (*P *<* *0.001 by Dunnett's *post hoc* test; Fig. 6B and *P* < 0.01 by Dunnett's *post hoc* test; Fig. 6C). Therefore, in addition to PI3K, AKT is indispensable for enhancement of Na^+^ transport by insulin. To verify an involvement of AKT in the insulin pathway we analyzed the phosphorylation of AKT with Western Blot. Although the total amount of AKT was not altered in insulin‐treated FDLE cells compared to controls, the amount of phosphorylated AKT was almost doubled after incubation with 200 nmol/L insulin (Fig. 6D and E). Since phosphorylation of AKT at Thr308 is an indicator for its activation, the results show an induction of AKT by insulin treatment in FDLE cells.

**Figure 7:**
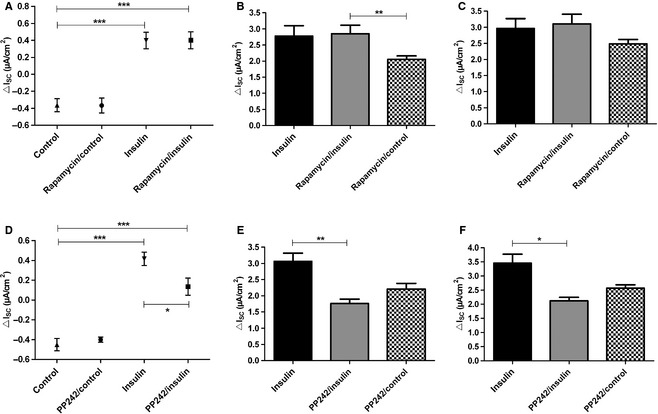
Inhibition of mTORC1 did not affect insulin‐induced *I*_SC_ increase, whereas inhibition of mTORC2 reduced insulin‐induced *I*_SC_ increase. Inhibition of the insulin‐induced response by Rapamycin (A–C) and PP242 (D–F). Rapamycin (100 nmol/L) or PP242 (1 *μ*mol/L) were added 30 min prior to addition of 200 nmol/L insulin to both compartments (*n *=* *11/7). For comparison 200 nmol/L insulin without Rapamycin/PP242 (*n *=* *13/19) and unstimulated control monolayers with Rapamycin/PP242 (*n *=* *12/8) were used as control. (A) insulin‐induced Δ*I*_SC_ in the presence of Rapamycin. Mean ± SEM, ***P <* *0.001. (B) *I*_amil_ in the presence of Rapamycin, **P <* *0.01. (C) *I*_ouab_ in the presence of Rapamycin. (D) insulin‐induced Δ*I*_SC_ in the presence of PP242. Mean ± SEM, ***P <* *0.001, *P <* *0.05. (E) *I*_amil_ in the presence of PP242, **P <* *0.01. (F) *I*_ouab_ in the presence of PP242, *P <* *0.05. Mean + SEM. 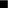, Insulin; 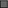, insulin + Rapamycin/PP242 ; 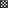, control + Rapamycin/PP242.

Aside from PI3K, mTORC1 is a suggested mediator of the insulin pathway. We used Rapamycin as inhibitor of mTORC1, to exclude an involvement of mTORC1 in the stimulation of Na^+^ transport by insulin. In Ussing chamber measurements, the inhibition of mTORC1 by incubation with Rapamycin (100 nmol/L) for 30 min prior to the addition of 200 nmol/L insulin had no effect on stimulation of *I*_SC_ by insulin (Fig 7A). Monolayers treated with insulin did not differ from Rapamycin‐/insulin‐treated monolayers. The analysis of *I*_amil_ (Fig. 7B) and *I*_ouab_ (Fig. 7C) showed that currents of insulin and Rapamycin‐/insulin‐treated monolayers were almost identical.

Since phosphorylation of AKT and SGK1 by mTORC2 is essential for their full activity, we investigated the effect of PP242 on insulin stimulation. In Ussing chamber measurements the addition of PP242 (1 *μ*mol/L) 30 min prior to addition of insulin, partly blocked the insulin effect in FDLE cells (Fig. 7D). Moreover, *I*_amil_ and *I*_ouab_ were decreased by PP242 (*P *<* *0.01 by Dunnett's *post hoc* test; Fig. 7E and *P* < 0.05 by Dunnett's *post hoc* test; Fig. 7F). These results suggest that mTORC2 is involved in the PI3K‐dependent pathway leading to activation of ENaC.

Taken together, the analysis of results obtained with blockers of intracellular mediators showed a clear dependency of insulin on the function of AKT/PI3K and mTORC2 to stimulate epithelial Na^+^ transport.

**Figure 8:**
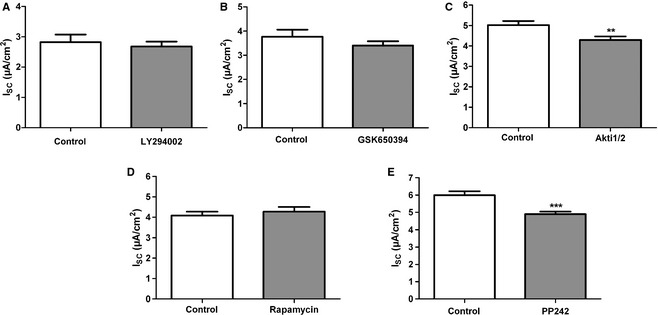
Effect of insulin on basal *I*_SC__._ Basal *I*_SC_ in the presence of different inhibitors compared to control monolayers. (A) LY‐294002. (B) GSK650394. (C) Akti1/2, **P <* *0.01. (D) Rapamycin. (E) PP242, ***P <* *0.001. Mean + SEM. 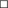, control; 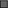, kinase inhibitor.

In addition to the stimulatory effects of insulin on *I*_SC_, we also examined basal currents and the effect of the kinase inhibitors on basal Na^+^ transport (Fig. 8). Inhibition of PI3K (Fig. 8A), SGK1 (Fig. 8B), and mTORC1 (Fig. 8D) had no effect on the basal *I*_SC_. However, the inhibitors of AKT (Fig. 8C) and mTORC2 (Fig. 8E) significantly reduced basal *I*_SC_.

### Involvement of the IGF‐1R/IR

**Figure 9:**
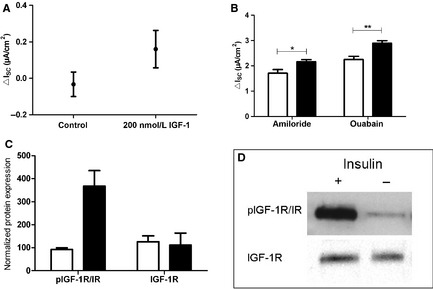
IGF‐1 increases *I*_SC._ Effect of 200 nmol/L IGF‐1 on *I*_SC_ in FDLE cells (*n *=* *6) compared to controls (*n *=* *9). (A) IGF‐1‐induced Δ*I*_SC_. Mean ± SEM. (B) *I*_amil_ and *I*_ouab_. Mean + SEM, *P <* *0.05, **P <* *0.01. (C) and (D) Western Blot analysis of IGF‐1R and pIGF‐1R/IR in FDLE cells grown in the presence of 200 nmol/L insulin compared to unstimulated controls. (C) Normalized densitometric evaluation of pIGF‐1R/IR and IGF‐1R. (D) Western Blot of pIGF‐1R/IR and total IGF‐1R resulted in 95 kDa bands (*n *=* *3). 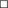, control; 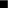, insulin

Since insulin is capable of activating the IGF‐1R in addition to the insulin receptor, we analyzed the effect of IGF‐1 on epithelial Na^+^ transport. The addition of 200 nmol/L IGF‐1 to Ussing chambers revealed a small increase in Δ*I*_SC_ (Fig. 9A) and a significant increase in *I*_amil_ and *I*_ouab_ (*P *<* *0.05 and *P *<* *0.01 by *T*‐test; Fig. 9B). The IGF‐1 concentrations of 2 nmol/L and 20 nmol/L did not significantly increase the *I*_SC_ (data not shown). To verify receptor activation, the rate of IGF‐1R/IR autophosphorylation was analyzed in Western Blots. After treating FDLE cells with 200 nmol/L insulin the total amount of IGF‐1R did not differ from unstimulated controls, whereas the phosphorylated IGF‐1R/IR was highly increased after insulin treatment (Fig. 9C and D).

## Discussion

This study elucidates pathways by which insulin stimulates alveolar epithelial Na^+^ transport. We demonstrate that PI3K and AKT mediate the stimulatory action on ENaC‐like amiloride‐sensitive channels in a mTORC2‐dependent manner. We also showed a concentration dependency of the insulin effect. These pathways differ from the mechanism by which insulin was shown to stimulate amiloride‐insensitive ion transport mechanisms (Hagiwara et al. 1992), which were inhibited by lavendustin A and attributed to a PKA‐dependent mechanism. In addition, the *I*_SC_ increase induced by insulin differs from the stimulating effect detected by other groups (Deng et al. 2012) using RT‐PCR and Western Blot, because the *I*_SC_ stimulation occurred within minutes after insulin addition. The determination of the apical and basolateral Na^+^ permeability showed that insulin increases the amiloride‐sensitive apical Na^+^ transport, whereas the Na,K‐ATPases was not affected. Since it has been shown previously that the *β*_1_‐subunit is rate‐limiting for the assembly of the Na,K‐ATPases (Chow and Forte 1995; Thome et al. 2001), insulin‐promoted translocation of the Na,K‐ATPase *α*_1_‐subunit to the plasma membrane (Comellas et al. 2010) may not result in an increase in functional Na,K‐ATPases. Therefore, our permeabilization studies do not contradict the results about insulin‐dependent translocation of Na,K‐ATPase *α*_1_‐subunits (Comellas et al. 2010). Furthermore, we demonstrate that insulin stimulated highly selective Na^+^ channels, whereas nonselective Na^+^ channels were not affected.

Since the increase in *I*_SC_ developed within a few minutes, we expected intracellular mediators rather than transcriptional alterations as the stimulatory pathway. PI3K is an important mediator of the insulin effect (Blazer‐Yost et al. 2003). It was shown that insulin stimulation induces migration of ENaC to the cell membrane in renal cells, which is critically dependent on PI3K activity (Blazer‐Yost et al. 2003). The inhibition of PI3K with LY‐294002 altered colocalization of ENaC and PI3K and thereby prevented translocation of ENaC to the cell membrane (Blazer‐Yost et al. 2003). Our results support the dependency of insulin on PI3K to stimulate Na^+^ transport. In taste receptor cells an increase in amiloride‐sensitive Na^+^ currents was shown after adding insulin using whole cell patch clamp recordings and ratiometric Na^+^ imaging (Baquero and Gilbertson 2011). Moreover, the effect of insulin on amiloride‐sensitive Na^+^ currents was abolished by addition of LY‐294002, whereas basal amiloride‐sensitive Na^+^ currents were not affected by LY‐294002 application (Baquero and Gilbertson 2011), which is in line with our results. In adult ATII cells insulin was shown to increase mRNA‐ and protein expression of *α*‐, *β*‐ and *γ*‐ENaC after 2 h incubation, which was prevented by LY‐294002 treatment (Deng et al. 2012). However, our Ussing chamber measurements suggest an additional much faster response to insulin which is unlikely to result from increased transcription. Therefore, several pathways seem to account for the different phases of PI3K‐dependent insulin stimulation of Na^+^ transport. Taken together, compelling evidence speaks for an involvement of PI3K in the effect of insulin on ENaC as demonstrated in cells from renal, tongue, and respiratory origin (Blazer‐Yost et al. 2003; Baquero and Gilbertson 2011; Deng et al. 2012), that also appears to account for increased ENaC function in FDLE cells, as shown by our results.

LY‐294003 is a commonly used inhibitor of PI3Ks, however, it is also reported to inhibit other kinases such as mTORC1 (Bain et al. 2007). To exclude an involvement of mTORC1, the specific mTORC1‐inhibitor Rapamycin was used. Since inhibition of mTORC1 did not diminish the stimulating effect of insulin, the results suggest that mTORC1 is not involved in ENaC regulation by insulin in FDLE cells, which is in line with earlier studies (Mansley and Wilson 2010a). In addition, it was also demonstrated, that LY‐294002 does not influence the insulin effect by inhibiting mTORC1.

SGK1 is also suggested to be a mediator of insulin effect in thyroid, respiratory and renal cells (Lee et al. 2007; Mansley and Wilson 2010b; Deng et al. 2012). In Western Blot measurements, we showed that insulin does activate SGK1 in FDLE cells, however, the inhibition of SGK1 does not prevent the stimulatory effect of insulin on Na^+^ transport, as shown by inhibition of SGK1 by GSK650394 in Ussing chamber measurements. This finding is different from mpkCCD cells, where insulin increased the *I*_SC_ in a SGK1‐dependent manner (Mansley and Wilson 2010b). We cannot exclude that culture conditions might be responsible for the different results since the mpkCCD cells were grown in hormone‐deprived media and our FDLE cells were cultured with serum supplementation. On the other hand, different cell types might show different reactions to insulin. For example, a study of the respiratory cell line H441, supplemented with dexamethasone, showed no increased phosphorylation of NDRG1 after incubation with insulin (Wilson et al. 2010). In renal mpkCCD cells, however, the same study showed that the effect of insulin was critically dependent on activation of SGK1 (Wilson et al. 2010). This suggests different regulatory mechanisms for channel activity between different cell types. Taken together, we conclude that SGK1 does not play an important part in the rapid stimulating effect of insulin on Na^+^ transport in FDLE cells. Nevertheless, we showed an increased activation of SGK1 after incubation with insulin. Therefore, a role of SGK1 in regulation of cell functions might be possible in FDLE cells, but at least was not detectable by Ussing chamber measurements within the time frame of our experiments. Transcriptional regulation of ENaC channels, on the other hand, might be regulated at least in part by SGK1, since in adult ATII cells inhibition of SGK1 and AKT reduced insulin‐induced increase in ENaC mRNA and protein expression to a higher extent than the application of an AKT inhibitor alone (Deng et al. 2012). However, sole inhibition of AKT was able to reduce the ENaC level of insulin‐stimulated cells to the control level, whereas the SGK1 inhibitor was not tested alone, only in combination with the AKT inhibitor (Deng et al. 2012). Taken together, an additional contribution for SGK1 in ENaC expression in alveolar cells might be possible, which, however, appears to work on a different time scale, typical for transcriptional effects, which might be too slow to be observed functionally in our electrophysiological measurements. Furthermore, our data in comparison with studies in adult ATII cells (Deng et al. 2012) indicate that the contribution of different regulatory pathways might change during ontogenic development, that is that FDLE cells use different pathways than adult ATII cells. Earlier findings demonstrated that levels of ENaC‐ and Na,K‐ATPase‐subunits are differentially regulated throughout development (Orlowski and Lingrel 1988; Tchepichev et al. 1995; Banasikowska et al. 2004). Besides, insulin levels vary throughout ontogenesis (Mitanchez 2008). Furthermore, we provide measurements of real transport processes, which might not be proportional to alterations in transport protein expression.

Next, we determined the role of AKT in the stimulation of epithelial Na^+^ transport by insulin. In our electrophysiological analysis, the inhibition of AKT by Akti1/2 completely abolished the effect of insulin, which is in line with earlier findings on other cell types (Mansley and Wilson 2010b). It was shown that AKT increases ENaC activity by phosphorylation of Nedd4‐2, thereby reducing the affinity of Nedd4‐2 to ENaC (Lee et al. 2007). Akti1/2 is a highly selective noncompetitive inhibitor of AKT (Barnett et al. 2005), which prevents the conformational change, triggered by the binding of phosphatidylinositol 3,4,5‐bisphophat (PIP_3_) to the pleckstrin homology domain of AKT isoforms, that allows PDK1 and mTORC2 to phosphorylate and activate AKT (Bain et al. 2007). However, it has been reported that Akti1/2 might also inhibit SGK1, since it was shown to prevent phosphorylation of NDRG1 (Mansley and Wilson 2010b), even though others reported no inhibition of SGK1 with Akti1/2 at concentrations as high as 250 *μ*mol/L (Barnett et al. 2005). In our experiments, we assume that a possible unspecific effect of Akti1/2 is negligible, since direct inhibition of SGK1 by GSK650394 did not affect insulin stimulation. Therefore, the complete suppression of the insulin effect by Akti1/2 was attributable to the inhibition of AKT. In addition, Western Blot experiments showed increased phosphorylation and thus an activation of AKT after incubation with insulin. Furthermore, analysis of basal *I*_SC_ showed a dependency of unstimulated Na^+^ transport on AKT. Thus, in FDLE cells AKT seems to be the major regulatory kinase in basal and insulin‐stimulated Na^+^ transport. The half‐life for activation of AKT by insulin is 1 min (Alessi et al. 1996) and, therefore, lies within the time frame of the observed effects. Nevertheless, it is not known, if AKT directly interacts with ENaC or Nedd4‐2. A study using recombinant AKT in outside‐out Patch Clamp recordings of Xenopus laevis oocytes showed a rapid increase in ENaC open probability in single‐channel measurements (Diakov et al. 2010). This rapid response was dependent on a phosphorylation of S621 in the *α*‐subunit of ENaC. Since the unspecific phosphatase inhibitor okadaic acid mimicked this reaction, a direct phosphorylation by AKT was not proven. In addition, the same study showed a delayed response to AKT coexpression, which was independent of S621 phosphorylation of *α*‐ENaC. This delayed response was attributed to changes in channel trafficking (Diakov et al. 2010). Following this and other investigations, the regulation of ENaC is not completely understood. Additional mediators like phospholipids might also be involved through PI3K, not only for activating PDK1 and downstream AKT but also as direct regulators of ENaC activity (Ma et al. 2002; Pochynyuk et al. 2006). The anionic phospholipids phosphatidylinositol 4,5‐bisphosphate (PIP_2_) and PIP_3_ were shown to increase amiloride‐sensitive ENaC currents without affecting surface expression of ENaC (Ma et al. 2002). However, since the inhibition of AKT completely blocked the effect of insulin in our experiments, a direct regulation of ENaC by phospholipids following insulin stimulation is not likely.

PDK1 and mTORC2 are known to phosphorylate AKT and SGK1, therefore activating these kinases. Phosphorylation of Ser422 by mTORC2 induces further phosphorylation of Thr256 in SGK1 by PDK1 (Garcia‐Martinez and Alessi 2008). The first phosphorylation does not activate SGK1, whereas phosphorylation at Thr256 results in an activation of SGK1. Only the phosphorylation of both residues leads to full SGK1 activity (Garcia‐Martinez and Alessi 2008). Similarly, the phosphorylation of AKT at Ser473 by mTORC2, facilitates phosphorylation of Thr308 by PDK1 (Sarbassov et al. 2005). Using PP242 as an inhibitor of mTOR, we were able to show a dependency of insulin effects on mTORC2 in FDLE cells. PP242 also inhibits mTORC1, however, since the specific inhibitor of mTORC1, Rapamycin failed to suppress the insulin stimulation, mTORC2 remains as the only possible mediator. PP242 did not completely block, but only reduced the insulin effect on *I*_SC_, which is in line with previous findings. In another study of mTORC2‐deficient cells it was shown that AKT is still activated to a significant extent (Jacinto et al. 2006), as it is phosphorylated at Thr308 by PDK1 in a reaction that is not dependent upon mTORC2 (Garcia‐Martinez and Alessi 2008). Thus, the incomplete inhibition of the insulin effect by PP242 is well in line with a stimulation via PI3K/AKT way. Aside from AKT, mTORC2 is known to phosphorylate SGK1. Since we could not show a rapid effect on ENaC activity by SGK1 in Ussing chamber measurements, we do not assume that the PP242 effect was achieved by inhibiting the activity of SGK1. Rather, we conclude that the suggested pathway of insulin via PI3K/AKT is dependent on mTORC2, which is permissive for the insulin effect in FDLE cells. Aside from insulin‐stimulated *I*_SC_, mTORC2 also influences the basal *I*_SC._ This finding indicates a role of mTORC2 on ENaC activity independent of insulin stimulation.

The effect of insulin was mimicked by IGF‐1, which also stimulated Na^+^ transport in electrophysiological studies. In Western Blot measurements, the insulin stimulation resulted in an increased autophosphorylation of the IGF‐1R/IR. Therefore, the effect of insulin might be mediated at least in part by binding of insulin to the IGF‐1R. However, since the antibodies employed in this study do not distinguish between phosphorylation of IGF‐1R, insulin receptor or possible IGF‐1/insulin hybrid receptors a determination of the direct contribution of each receptor type is not possible. In toad urinary bladder cells, insulin and IGF‐1 were both able to increase Na^+^ transport which was inhibited by amiloride (Blazer‐Yost et al. 1989). These results led to the assumption that ligands binding to specific insulin receptors and IGF‐1R stimulate Na^+^ transport, while insulin and IGF‐1 activated pathways might be identical or converge following ligand binding (Blazer‐Yost et al. 1989). Therefore, we assume that the effect of insulin in FDLE cells is mediated through binding of the insulin receptor and the IGF‐1R and similar pathways are activated by both ligands.

In summary, we determined the effect of insulin on Na^+^ transport as activation of apical highly selective Na^+^ channels, in FDLE cells, which was dependent on the activity of PI3K and AKT, but not on the activity of SGK1. We suggest a pathway of insulin action via IGF‐1R/IR, leading to activation and autophosphorylation of the receptors. Activation of receptor tyrosine kinases results in activation of PI3K and subsequently forming of phospholipids, leading to activation of PDK1 and mTORC2. These kinases phosphorylate and activate AKT, resulting in an increased epithelial Na^+^ transport. Our results differ from, but do not contradict earlier findings, suggesting SGK1 as the main kinase in ENaC activation by insulin in other cell types and with different time frames (Mansley and Wilson 2010b). Several different mechanisms may be involved in regulation of existing channels and transcription of channel genes. We assume different ways of activation of ENaC by SGK1 and AKT, as several groups showed additional effects of both kinases (Lee et al. 2008; Deng et al. 2012). There are several regulatory mechanisms known for the insulin effect on ENaC, like translocation from intracellular pools to the plasma membrane (Blazer‐Yost et al. 2003; Tiwari et al. 2007), preventing degradation by phosphorylation of Nedd4‐2 (Lee et al. 2007) and activating the channel by direct phosphorylation (Shimkets et al. 1998; Zhang et al. 2005; Diakov et al. 2010) leading to an increased open probability (Tallini and Stoner 2002; Pavlov et al. 2013). Aside from that, basal control of ENaC function is strictly dependent on hormone levels, salt and water homeostasis and *in vivo* on stress and environmental conditions. Taken together, the conclusion that AKT and not SGK1 is most important for ENaC regulation by insulin in FDLE cells is surprising, but explicable and shows the importance of further investigations of ENaC regulation.

## Grants and Disclosures

No grants or conflicts of interest, financial or otherwise, are declared by the authors.

## Conflict of Interest

None declared.
