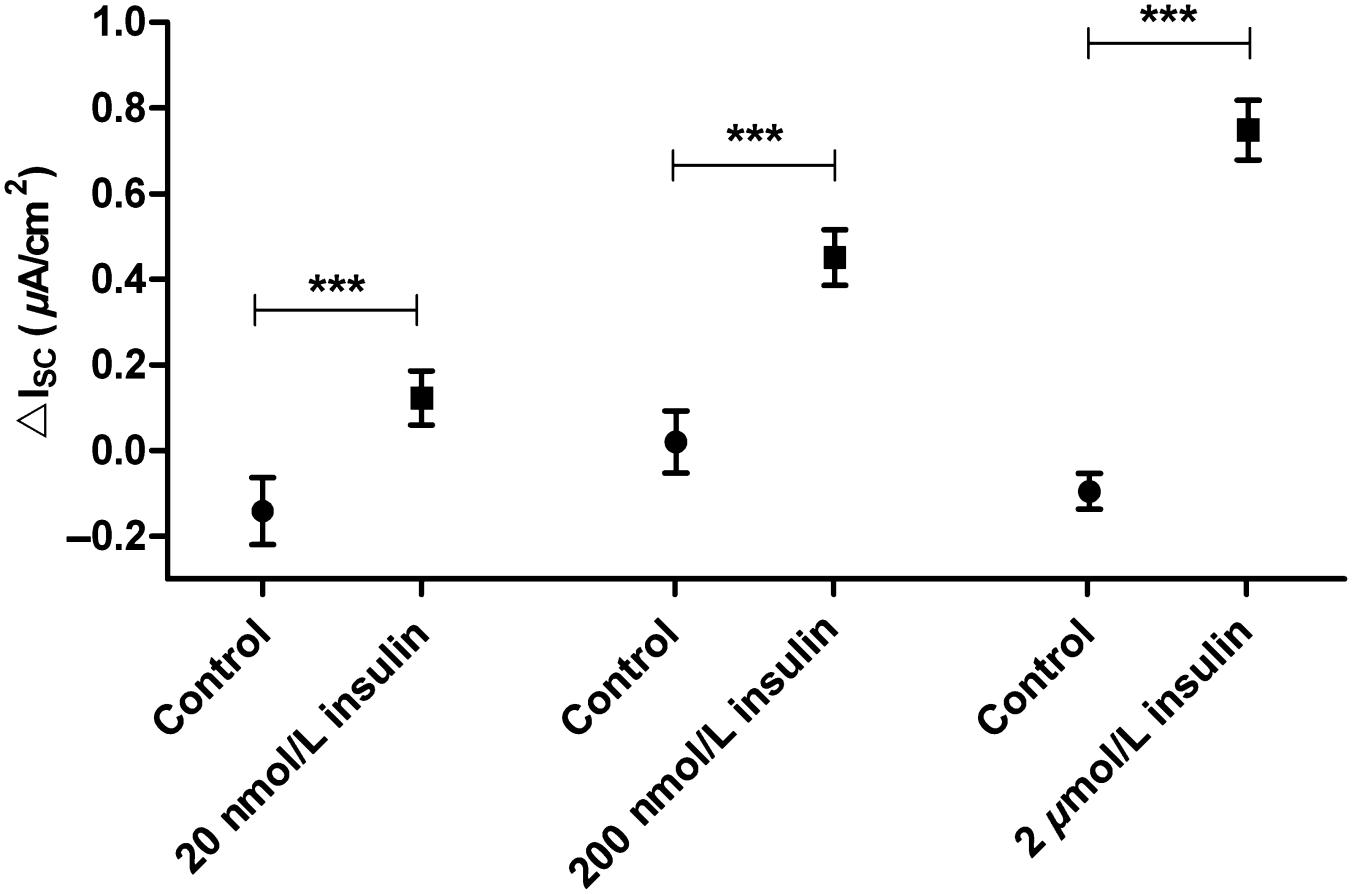# Erratum

**DOI:** 10.14814/phy2.12005

**Published:** 2014-04-23

## Introduction

**Rapid elevation of sodium transport through insulin is mediated by AKT in alveolar cells**

**Charlott Mattes, Mandy Laube & Ulrich H. Thome**

*Physiol Rep*, 2 (3), 2014, e00269, doi: 10.1002/phy2.269

In figure 1, the label “20 *μ*mol/L” has been corrected to “20 nmol/L insulin”, and “200 *μ*mol/L insulin” has been corrected to “200 nmol/L insulin”.

Please refer to the revised figure 1 below.